# Comparison between topical cetirizine with minoxidil versus topical placebo with minoxidil in female androgenetic alopecia: a randomized, double-blind, placebo-controlled study

**DOI:** 10.1007/s00403-022-02512-2

**Published:** 2022-12-26

**Authors:** Eglal A. Bassiouny, Solwan I. El-Samanoudy, Maggie M. Abbassi, Hanan R. Nada, Samar F. Farid

**Affiliations:** 1 Department of Clinical Pharmacy, Faculty of Pharmacy, Cairo University, Al Kasr Al Ainy Street, Cairo, Egypt; 2 Department of Dermatology, Faculty of Medicine, Cairo University, Al Kasr Al Ainy Street, Cairo, Egypt

**Keywords:** Cetirizine, Alopecia, FAGA, Hair thickness, Patient self-assessment, Global photographic assessment

## Abstract

Androgenetic alopecia (AGA) is the most common cause of hair loss in both genders with a higher psychological impact on females. Currently, topical minoxidil is the only FDA-approved treatment for female AGA and it needs life-long application and causes side effects. Cetirizine is an antihistamine that may be effective in hair loss treatment. This study aimed to compare the efficacy and safety of topical cetirizine with minoxidil (group 1) versus topical minoxidil with placebo (group 2) in female patients with AGA. This was a double-blind, randomized, controlled, parallel study conducted at Dermatology Clinic, Cairo University Teaching Hospital (Kasr- Al- Ainy), Egypt. Sixty-six patients with female AGA, aged 20–50 years, Sinclair (II–IV), were randomly assigned to one of the 2 groups for 24 weeks. The trichoscopic parameters, patients’ self-assessment, side effects and global photographic assessment were evaluated. There was a statistically significant change from baseline in frontal and vertex terminal and vellus hair density (*P* < 0.0005) with a significant increase in vertex hair shaft thickness and average number of hairs per follicular unit in group 1 (*P* < 0.05). Patients reported significantly better scores in patient self-assessment in group 1 (*P* < 0.05). Side effects were not significantly different between groups (*P* > 0.05). Topical cetirizine increases hair shaft thickness and results in a higher clinical improvement from patients’ perspective with a good safety profile (NCT04481412, study start date: July 2020).

## Introduction

Androgenetic alopecia (AGA) is the most common cause of hair loss in both genders [1]. It is a hereditary, androgen-dependent, age-dependent condition with variable onset [2]. Hair loss process involves progressive miniaturization of androgen sensitive hair follicles associated with lymphocytic infiltrate and high level of prostaglandin D2 (PGD2) that inhibits hair growth [3, 4]. The role of androgens in female androgenetic alopecia (FAGA) is not completely understood, and its response to antiandrogens is unpredictable [5, 6]. Women with AGA suffer more from social withdrawal compared to men [7–9]. Currently, the FDA-approved treatment for FAGA is topical minoxidil which needs lifelong application and has side effects [10–12]. Cetirizine is a well-tolerated antihistamine with anti-inflammatory effect, reduces PGD2 that inhibits hair growth and increases PGE2 that promotes it [13–15]. Few studies were conducted on cetirizine in AGA, but they showed contradictory results [15–17]. Based on the above evidence and lacking studies, this study was conducted to evaluate the safety and efficacy of topical cetirizine in FAGA.

## Patients and methods

### Study design and study participants

This was a 24-week, double-blind (patient and outcome assessors), randomized, controlled, parallel study. Sixty-six patients were recruited from the Dermatology Clinic, Cairo University Teaching Hospital (Kasr- Al- Ainy), Egypt, after obtaining their written consent and after the approval of the Research Ethics Committee for Experimental and Clinical Studies, Faculty of Pharmacy, Cairo University (CL 2555). The study was registered in clinicaltrials.gov, identifier number NCT04481412.

The inclusion criteria were: FAGA patients aged 20 to 50 years, experiencing active hair loss within the last year, Sinclair grade of II-IV, willing to continue their current regimen of vitamins and not start any new vitamins and to use a mild non-medicated shampoo and conditioner during the study, did not receive any topical or systemic treatment for AGA or PGs in the last 6 months. The exclusion criteria were: patients with other chronic dermatological conditions, hair transplants, scalp reduction, current hair weave or tattooing in the target area, received radiation therapy to the scalp, or had chemotherapy in the past year, had a known underlying medical problem that could influence hair growth, with clinical diagnosis of non-AGA forms of alopecia, pregnant or lactating females or planning to become pregnant during the study, with severe cardiovascular disease, with hair loss for greater than 5 years, with known hypersensitivity to any of the treatments components, using medications that induce hair loss within the last 3 months and any patients refusing to participate.

### Randomization

Patients were randomly assigned into 2 groups with a 1:1 allocation. A blocked size balanced randomization was done through the free online software “sealed envelope.” The software randomly generated numbers that indicated the specific group to which the research subject would be allocated [18]. The sequence of the generated numbers with its indicated specific group were kept hidden from the investigator till the patient enrollment interview finished and interventions were assigned. Each group had 33 patients, and both of them applied 1 ml of topical minoxidil (5%) once daily in the morning for 24 weeks. In addition, group 1 applied 1 ml of topical cetirizine (1%) and group 2 1 ml of placebo once daily in the evening. The 1% cetirizine solution was prepared by the investigator using 1% cetirizine in 96% ethanol solution. The placebo was made of 96% ethanol solution. Both were filled in identical bottles with identical labels. Patients were contacted on the 6th and the 18th weeks on the phone to check for side effects, compliance, stress on adherence and fill the self-assessment questionnaire. They were asked to bring the empty bottles at the next visit.

### Measurements

#### Primary outcomes

The changes from baseline in the terminal and vellus hairs percent and density, terminal to vellus ratio, mean hair shaft thickness and the average number of hairs per follicular unit in the frontal and vertex areas were measured at 12 and 24 weeks. Dermatoscopic examination was done using a digital dermatoscope (phototrichoscopy by FotoFinder, medicam 1000 Video dermoscopy, FotoFinder Systems GmbH, Germany, Trichoscale software, smart count mode-manual analysis) [19]. We used the contact polarized mode in this study (20 × to confirm clinical diagnosis and 50 × for trichoscopic measurements). Vellus hair is defined as any hair with a diameter < 30 µm, while terminal hair is any hair with a larger diameter [20].

Patient self-assessment was done every 6 weeks using predetermined 5 questions in Arabic, each of which evaluated a specific aspect of their hair compared with baseline. The questions asked about the change in hair elongation rate, new hairs, bald spots size, hair loss rate and self-satisfaction. Each of the first four questions was answered using a 7-point scale. The choices ranged from − 3 = greatly decreased to 3 = greatly increased. The fifth question was answered using a 5-point scale. The choices ranged from − 2 = very unsatisfied to 2 = very satisfied. The questions were inspired from the women’s hair growth questionnaire (WHGQ) [21, 22] with few modifications. A question about the patient satisfaction and the scales for items were added as above. Face validity was conducted by colleagues in the Clinical Pharmacy Department, Faculty of Pharmacy and the Dermatology Department, Faculty of Medicine, Cairo University.

#### Secondary outcomes

Global photographic assessment was conducted at baseline, 12 and 24 weeks at the clinic where standardized photographs of the scalp were taken. Then, a panel of 3 blinded dermatologists and the blinded outcome assessor compared baseline with follow-up photographs of each subject and used a 7-point scale to rate the hair growth. The choices ranged from − 3 = greatly decreased to 3 = greatly increased.

Treatment safety was evaluated by questioning patients every 6 weeks if they had suffered from any side effects since the last interview. Patients were informed about minoxidil side effects, and if any serious side effect had occurred, patients would have been withdrawn from the study and treated for free.

### Statistical analysis

A priori sample size was calculated using G*Power 3.1.9.2 using the trichoscopic evaluation parameters as the primary outcome measure. A total sample size of (28) participants would be required to detect a statistically significant change using both mixed repeated measures ANOVA (RM-ANOVA) and one-way RM-ANOVA tests predicting a medium effect size of 0.25, 2 sided alpha error = 0.05, power = 80% (*β* = 0.2). We decided to increase the total sample size to 66 to account for an expected 10% drop out rate [23].

Statistical Package for Social Science (SPSS) version 23 (SPSS Inc., Chicago, IL, USA) was used. Qualitative data were presented as numbers and percentages, whereas quantitative data were presented as mean and standard deviation. Student’s *t*-test was used to compare baseline numerical variables and the change from baseline between groups. Chi-square, Fisher’s exact and Mann–Whitney *U* tests were used to compare categorical variables.

One-way RM-ANOVA was applied to test the effect of time on numerical variables within each group individually. The mixed model of RM-ANOVA was applied to account for the time and group effect and their interaction. That was followed by pairwise comparisons with Bonferroni adjustments.

The patients’ responses to the self-assessment questionnaire and the results of the outcome assessor and the dermatologists’ panel evaluation were compared between the 2 groups at 6- and 12-week intervals, respectively, using Mann–Whitney *U* test, exact significance. The patients’ responses to the self-assessment questionnaire were compared within each group at 6-week intervals using Friedman test (exact significance). Post hoc analysis with Wilcoxon signed-rank tests was conducted with a significance level set at *P* < 0.005, exact significance.

Also, the percent of subjects that reported adverse effects was compared between groups using Fisher’s exact test. All tests were two-tailed at *α* = 0.05.

## Results

### Patient screening and baseline characteristics

**Fig. 1:**
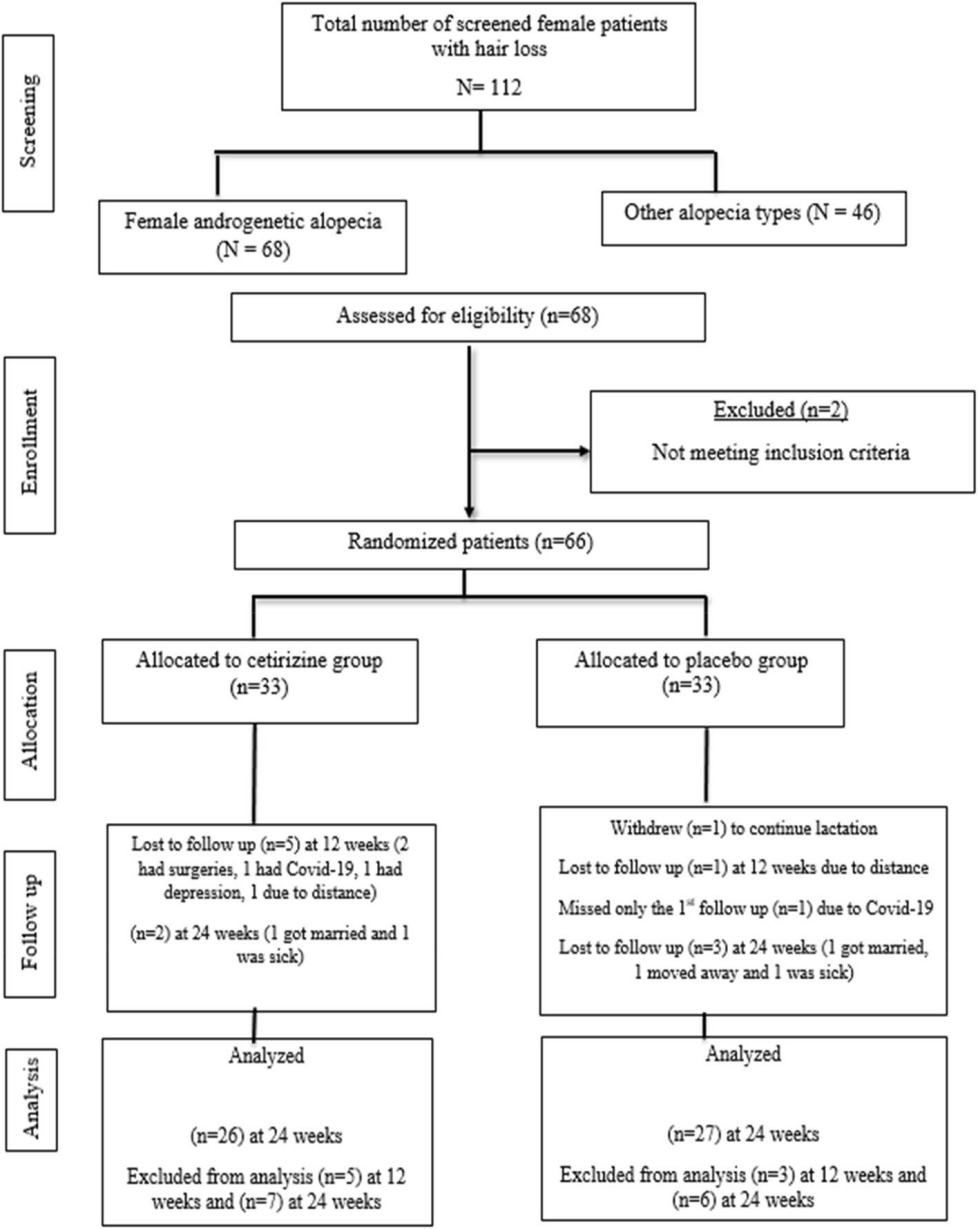
Flow chart of patients’ screening, enrollment, allocation, follow-up and analysis

The recruitment started from July 2020 till June 2021, and the follow-up ended in November 2021. Out of 66 patients, only 53 were included in the final analysis (Fig. 1). Some of the patients who dropped out filled the patient self-assessment questionnaire over the phone if they were still compliant to treatment. We conducted per protocol analysis. Patients’ demographics and baseline characteristics were comparable between groups (Table 1).

**Table 1:** Patients’ demographic data and baseline characteristics, expressed as mean ± SD or number (percentage) or mean rank

Parameter	Cetirizine + minoxidil group (*N* = 26)		Placebo + minoxidil group (*N* = 27)	*P*-value (< 0.05)
Age (years)	38.61 ± 8.74		36.74 ± 9.84	0.467^a^
Duration of hair loss (years)	3.19 ± 1.46		3.29 ± 1.87	0.823^a^
Sinclair grade				0.219^b^
II	9 (34.6%)		16 (59.3%)	
III	10 (38.5%)		7 (25.9%)	
IV	7 (26.9%)		4 (14.8%)	
Fertility				1.00^c^
Pre-menopause	23 (88.5%)		23 (85.2%)	
Post-menopause	3 (11.5%)		4 (14.8%)	
Family history	18 (69.2%)		21 (77.8%)	0.544^b^
Hirsutism	4 (15.4%)		4 (14.8%)	1.00^c^
Menstrual irregularities	8 (30.8%)		8 (29.6%)	1.00^b^
Acne	4 (15.4%)		8 (29.6%)	0.327^c^
**Frontal area trichoscopic parameters**				
Terminal hair percentage (%)	64.73 ± 15.63		70.11 ± 12.99	0.178^a^
Vellus hair percentage (%)	35.26 ± 15.63		29.88 ± 12.99	0.178^a^
Terminal hair density/cm^2^	97.62 ± 32.89		107.87 ± 28.63	0.231^a^
Vellus hair density/cm^2^	52.77 ± 23.91		46.92 ± 24.03	0.379^a^
Terminal:vellus	2.84 ± 3.39		3.21 ± 2.31	0.646 ^a^
Average thickness (µm)	42.29 ± 10.06		46.05 ± 8.93	0.155^a^
Average number of hairs/follicular unit	1.49 ± 0.22		1.56 ± 0.25	0.332^a^
**Vertex area trichoscopic parameters**				
Terminal hair percentage (%)	67.90 ± 13.63		71.74 ± 15.05	0.335^a^
Vellus hair percentage (%)	32.12 ± 13.60		28.25 ± 15.05	0.331^a^
Terminal hair density/cm^2^	104.75 ± 36.42		113.80 ± 33.07	0.348^a^
Vellus hair density/cm^2^	49.22 ± 23.25		43.15 ± 27.65	0.393^a^
Terminal:vellus	3.24 ± 3.98		4.17 ± 4.69	0.439 ^a^
Average thickness (µm)	43.81 ± 9.31		47.41 ± 10.32	0.188^a^
Average number of hairs/follicular unit	1.60 ± 0.33		1.63 ± 0.29	0.742^a^
**Patient self-assessment**	**Mean rank**			***P*-value** **(< 0.05)**
	(*N* = 27)		(*N* = 30)	
Hair growth	31.44		26.80	0.269^d^
New hairs	29.15		28.87	0.968^d^
Bald areas	31.06		27.15	0.262^d^
Hair loss	29.30		28.73	0.850^d^
Satisfaction	29.46		28.58	0.831^d^

^a^Student’s *t*-test, 2-tailed

^b^Chi-square test, 2-tailed

^c^Fisher’s exact test, 2-tailed

^d^Mann–Whitney *U* test, exact significance, 2-tailed

### Trichoscopic evaluation

**Fig. 2:**
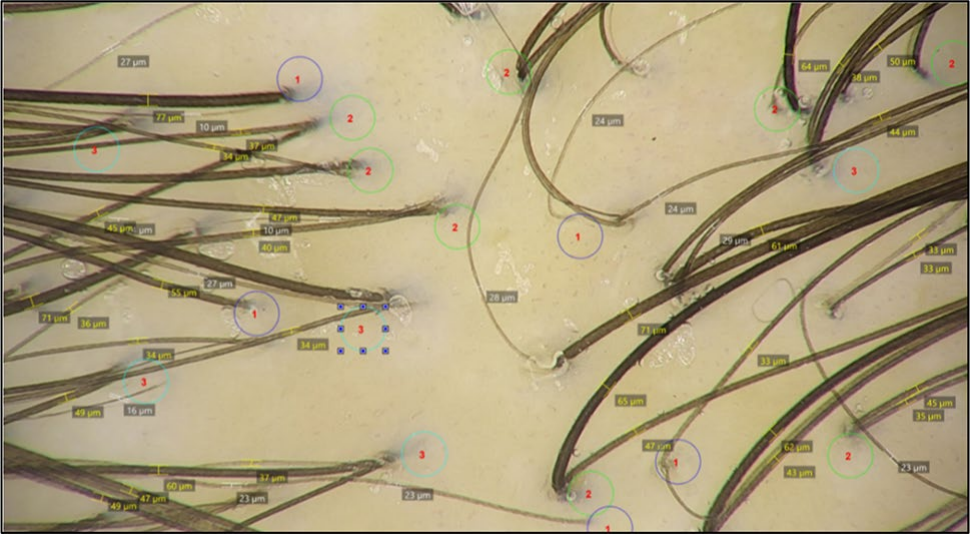
A trichocscopic image of a patient showing the hair count, thickness and follicular units using smart count mode (Fotofinder 50×, Trichoscale software)

Figure 2 shows the method used in calculating trichoscopic parameters by the Fotofinder smart count mode. The percentage of hairs as well as the total mean thickness were measured in a 50 × magnified field with an area of 0.238 cm^2^.

The frontal area showed a statistically significant increment in the terminal hair density between the three time points within both groups (*P* < 0.0005).

Post hoc test showed that the vellus hair density significantly increased from the baseline to the 24th week ((95% CI_density_, 21.1 to 55.2), *P* < 0.0005) and from the 12th week to the 24th week ((95% CI_density_, 18 to 47.6), *P* < 0.0005) but not from baseline to the 12th week within group 1. Also, in group 2, the vellus hair density significantly increased from the baseline to the 24th week ((95% CI_density_, 17.2 to 60.7), *P* < 0.0005) and from the 12th to the 24th week ((95% CI_density_, 5.1 to 48.4), *P* < 0.05) but not from baseline to the 12th week.

The vertex area showed a statistically significant increment in the terminal hair density between the three time points within both groups (*P* < 0.0005).

Post hoc test showed that the vellus hair density significantly increased from the baseline to the 24th week ((95% CI_density_, 14.6 to 62), *P* < 0.005) and from the 12th to the 24th week ((95% CI_density_, 18.7 to 59.2), *P* < 0.0005) but not from baseline to the 12th week within group 1. Also, in group 2, the vellus hair density significantly increased from the baseline to the 24th week ((95% CI_density_, 16.8 to 59.2), *P* < 0.0005) and from the 12th to the 24th week ((95% CI_density_, 7.9 to 49.3), *P* < 0.005) but not from baseline to the 12th week.

Also, in both areas, there was a statistically non-significant increase in the terminal hair percentage and decrease in the vellus hair percentage from baseline, but the change was slightly higher in group 1. The terminal-to-vellus hair ratio increased insignificantly from the baseline in group 1 and insignificantly decreased in group 2.

Regarding hair shaft thickness, there was a slight insignificant change within both groups in the frontal area. In the vertex area, the shaft thickness differed significantly within group 1 between time points (*P* = 0.046) and post hoc analysis showed significant difference between the baseline and the 12th week only ((95% CI, 0.2 to 6.7) µm, *P* = 0.029).

The average number of hairs per follicular unit changed significantly over time in group 1 in both the frontal and vertex areas (*P* = 0.007 and 0.008, respectively); post hoc analysis showed significant difference only between the baseline and the 12th week in frontal and vertex areas ((95% CI, 0.03 to 0.3), *P* = 0.011 and (95% CI, 0.07 to 0.42), *P* = 0.004, respectively). (*P* < 0.05, one way RM-ANOVA, post hoc Bonferroni adjustment).

The time*group interaction in both scalp areas was insignificantly different between groups (*P* < 0.05, mixed RM-ANOVA) (Tables 2, 3).

**Table 2:** Change in trichoscopic parameters in the frontal area within and between groups

Trichoscopic parameter	Cetirizine + minoxidil group (*N* = 26) Mean ± SD				Placebo + minoxidil group (*N* = 27) Mean ± SD				
	Baseline	12 weeks	24 weeks	*P*-value (t)*	Baseline	12 weeks	24 weeks	*P*-value (*t*)*	*P*-value (*t* × *g*)*
Terminal hair percentage (%)	64.73 ± 15.63	67.71 ± 14.24	66.76 ± 13.44	0.355^a^	70.11 ± 12.99	70.25 ± 11.88	70.20 ± 12.05	0.998^a^	0.665^b^
Vellus hair percentage (%)	35.26 ± 15.63	32.28 ± 14.24	33.23 ± 13.44	0.355^a^	29.88 ± 12.99	29.74 ± 11.88	29.79 ± 12.05	0.998^a^	0.665^b^
Terminal hair density/cm^2^	97.62 ± 32.89	120.45 ± 36.73	177.43 ± 43.15	< 0.0005*^a^	107.87 ± 28.63	133.44 ± 35.73	197.36 ± 43.07	< 0.0005*^a^	0.549^b^
Vellus hair density/cm^2^	52.77 ± 23.91	58.12 ± 28.29	90.98 ± 42.77	< 0.0005*^a^	46.92 ± 24.03	59.08 ± 35.73	85.90 ± 40.86	< 0.0005*^a^	0.724^b^
Terminal:vellus	2.84 ± 3.39	3.21 ± 3.46	2.99 ± 3.60	0.378 ^a^	3.21 ± 2.31	3.02 ± 1.86	3.19 ± 2.54	0.916 ^a^	0.601^b^
Average thickness(µm)	42.29 ± 10.06	43.91 ± 8.53	42.37 ± 7.97	0.217^a^	46.05 ± 8.93	47.17 ± 9.38	45.12 ± 9.35	0.345^a^	0.843^b^
Average number of hairs/ follicular unit	1.49 ± 0.22	1.68 ± 0.34	1.53 ± 0.21	0.007*^a^	1.56 ± 0.25	1.73 ± 0.34	1.60 ± 0.28	0.062^a^	0.965^b^

^*^Level of significance *P* ˂ 0.05

^a^One-way RM-ANOVA (Time effect (*t*)) at *P* ˂ 0.05

^b^Mixed RM-ANOVA (time*group interaction (*t* × *g*)) at *P* ˂ 0.05

**Table 3:** Change in trichoscopic parameters in the vertex area within and between groups

Trichoscopic parameter	Cetirizine + minoxidil group (*N* = 26) Mean ± SD					Placebo + minoxidil group (*N* = 27) Mean ± SD				
	Baseline	12 weeks	24 weeks		*P*-value (*t*)*	Baseline	12 weeks	24 weeks	*P*-value (*t*)*	*P*-value (*t* × *g*)*
Terminal hair percentage (%)	67.90 ± 13.63	72.11 ± 12.81		70.34 ± 13.96	0.193^a^	71.74 ± 15.05	74.01 ± 13.80	72.05 ± 10.99	0.567^a^	0.728^b^
Vellus hair percentage (%)	32.12 ± 13.60	27.88 ± 12.81		29.65 ± 13.96	0.188^a^	28.25 ± 15.05	25.98 ± 13.80	27.98 ± 11.04	0.562^a^	0.718^b^
Terminal hair density/cm^2^	104.75 ± 36.42	123.35 ± 32.26		197.35 ± 42.88	< 0.0005*^a^	113.80 ± 33.07	142.17 ± 43.15	198.77 ± 42.85	< 0.0005*^a^	0.324^b^
Vellus hair density/cm^2^	49.22 ± 23.25	48.56 ± 24.08		87.58 ± 47.49	< 0.0005*^a^	43.15 ± 27.65	52.54 ± 34.20	81.21 ± 43.33	< 0.0005*^a^	0.466^b^
Terminal:vellus	3.24 ± 3.98	5.50 ± 8.13		4.14 ± 4.89	0.166 ^a^	4.17 ± 4.69	4.05 ± 2.85	3.19 ± 1.84	0.263 ^a^	0.174 ^b^
Average thickness(µm)	43.81 ± 9.31	47.33 ± 8.15		45.65 ± 9.97	0.046*^a^	47.41 ± 10.32	48.66 ± 9.55	47.11 ± 8.68	0.573^a^	0.445^b^
Average number of hairs/follicular unit	1.60 ± 0.33	1.85 ± 0.39		1.71 ± 0.22	0.008*^a^	1.63 ± 0.29	1.76 ± 0.29	1.77 ± 0.25	0.051^a^	0.235^b^

^*^Level of significance *P* ˂ 0.05

^a^One-way RM-ANOVA (Time effect (*t*)) at *P* ˂ 0.05

^b^Mixed RM-ANOVA (time*group interaction (*t* × *g*)) at *P* ˂ 0.05

**Fig. 3:**
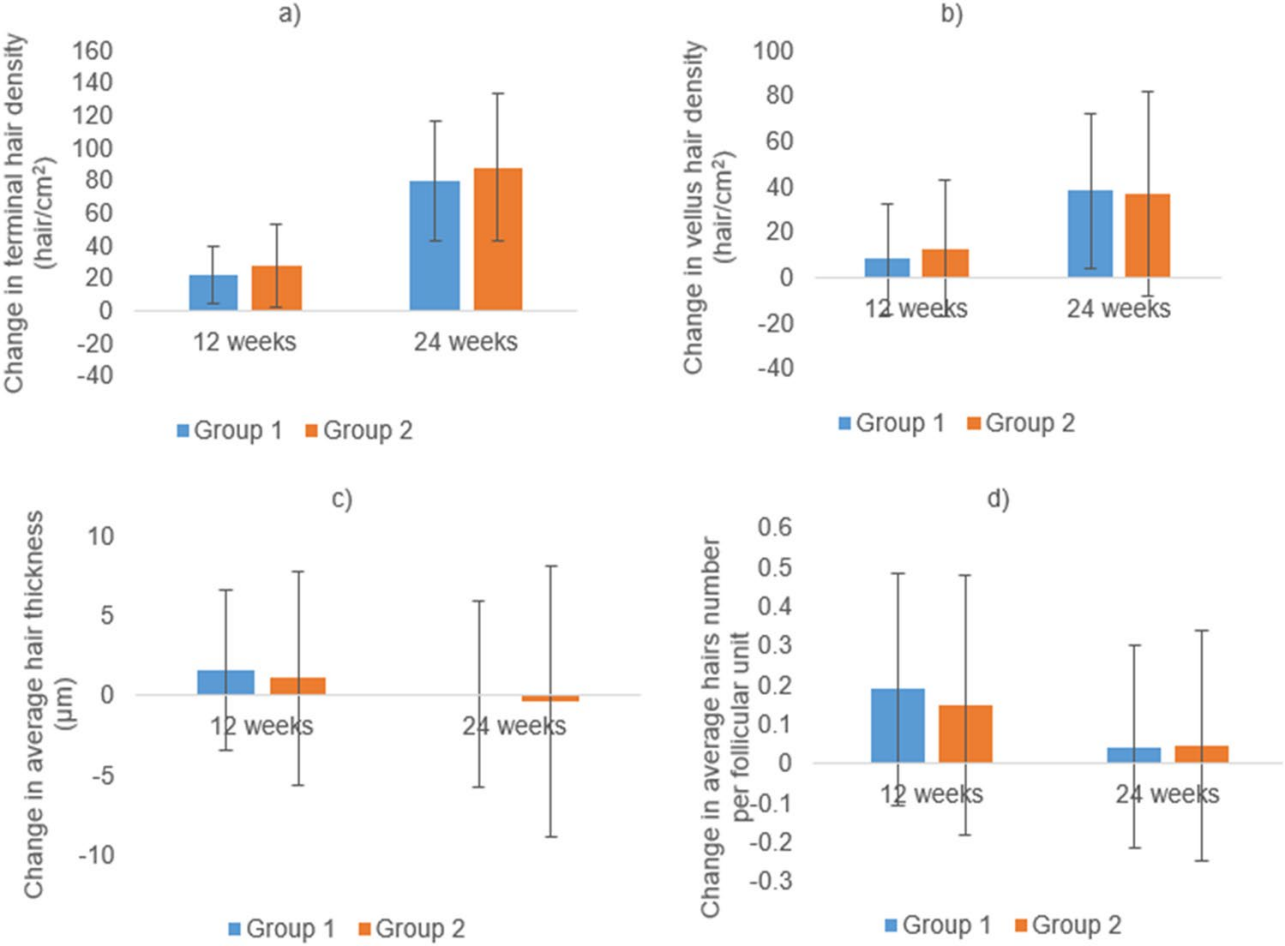
Changes from the baseline in trichoscopic parameters in the frontal area at 12 (*N* = 28 and 30) and 24 weeks (*N* = 26 and 28) of treatment in groups 1 (cetirizine) and 2 (placebo). **a** Terminal hair density, **b** vellus hair density, **c** average hair thickness, **d** average number of hairs per follicular unit. Insignificant difference between both groups at 12 and 24 weeks (Student’s *t* test)

**Fig. 4:**
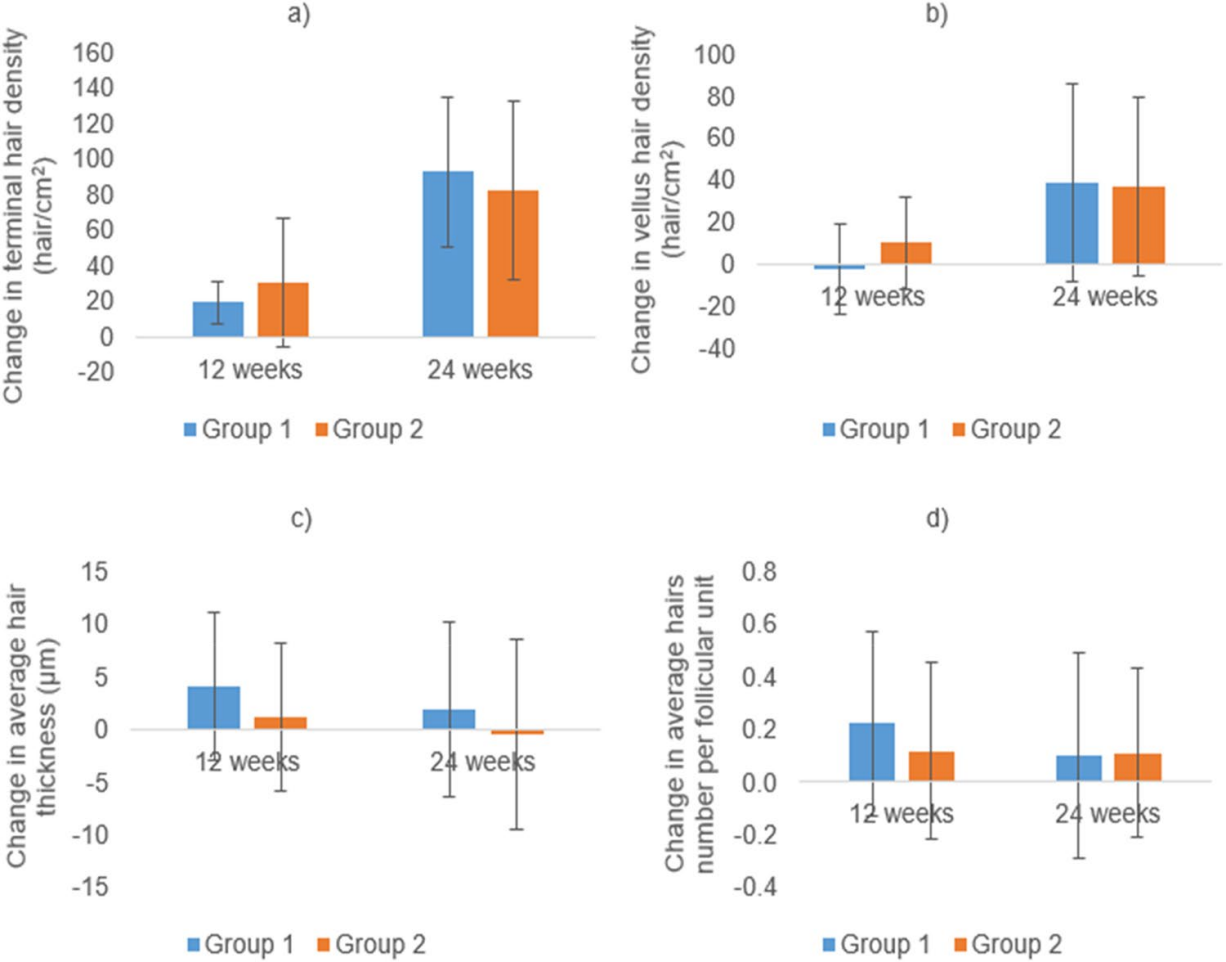
Changes from the baseline in trichoscopic parameters in the vertex area at 12 (*N* = 28 and 30) and 24 weeks (*N* = 26 and 28) of treatment in groups 1 (cetirizine) and 2 (placebo). **a** Terminal hair density, **b** vellus hair density, **c** average hair thickness, **d** average number of hairs per follicular unit. Insignificant difference between both groups at 12 and 24 weeks (Student’s *t* test) except for change in vellus hair density at 12 weeks (*P* = 0.034)

The changes from baseline in trichoscopic parameters were compared between groups at 12 and 24 weeks using Student’s *t*-test in both areas, but there was statistically insignificant difference between groups except for the vertex vellus hair density at the 12th week (*P* = 0.034) (Figs. 3, 4).

At 24 weeks, the relative risk ratio for decrease in hair thickness in group 1 compared to group 2 was 0.68 (95% CI 0.4–1.1) and 0.76 (95% CI 0.4–1.4) in the frontal and vertex areas, respectively. Although cetirizine showed a protective effect against decrease in hair thickness, the difference was statistically insignificant.

### Patient self-assessment questionnaire

Patients responses were compared between groups at 6 week-intervals using Mann–Whitney *U* test (exact significance, *P* < 0.05). At 6 weeks, group 1 was insignificantly better in all parameters except the hair growth, hair loss and patient satisfaction parameters. At 12 and 18 weeks, group 1 was significantly better in the new hairs parameter (*P* = 0.046 and 0.048, respectively) and insignificantly better in the other parameters. At 24 weeks, group 1 was insignificantly better in the hair loss parameter and significantly better in all other parameters (*P* < 0.05) (Tables 4, 5).

**Table 4:** Comparison of patients’ self-assessment between groups at 6 and 12 weeks of treatment

Patients self-assessment parameter	6 weeks				12 weeks			
	Cetirizine + minoxidil group (*N* = 32)	Placebo + minoxidil group (*N* = 32)	*U*- value	*P*-value* (< 0.05)	Cetirizine + minoxidil group (*N* = 32)	Placebo + minoxidil group (*N* = 31)	*U*- value	*P*-value* (< 0.05)
	Mean rank	Mean rank			Mean rank	Mean rank		
Hair growth	32.61	32.9	508.5	0.958	33.77	30.18	439.5	0.419
New hairs	35.28	29.72	423	0.217	36.17	27.69	362.5	0.046*
Bald areas	29.84	35.16	427	0.229	30.09	33.97	435	0.376
Hair loss	33.92	31.08	466	0.540	28.72	35.39	391	0.111
Satisfaction	32.16	32.84	501	0.883	34.47	29.45	417	0.172

^*^Mann–Whitney *U* test, exact significance, 2-tailed

**Table 5:** Comparison of patients’ self-assessment between groups at 18 and 24 weeks of treatment

Patients self-assessment parameter	18 weeks				24 weeks			
	Cetirizine + minoxidil group (*N* = 28)	Placebo + minoxidil group (*N* = 30)	*U*-value	*P*-value* (< 0.05)	Cetirizine + minoxidil group (*N* = 27)	Placebo + minoxidil group (*N* = 30)	*U*-value	*P*-value* (< 0.05)
	Mean rank	Mean rank			Mean rank	Mean rank		
Hair growth	31.61	27.53	361	0.365	33.81	24.67	275	0.030*
New hairs	33.77	25.52	300.5	0.048*	36.35	22.38	206.5	< 0.0005*
Bald areas	27.95	30.95	376.5	0.474	23.63	33.83	260	0.012*
Hair loss	27.09	31.75	352.5	0.230	26.06	31.65	325.5	0.082
Satisfaction	31.27	27.85	370.5	0.341	33.50	24.95	283.5	0.026*

^*^Mann–Whitney *U* test, exact significance, 2-tailed

Patients’ responses were compared within each group at 6-week intervals using Friedman test (*P* < 0.05, exact significance), and all parameters significantly improved within each group over time (Table 6). Post hoc analysis (Wilcoxon signed rank test, *P* < 0.005) showed statistically significant differences between all the time points in all parameters in group 1 except for the following; there was insignificant difference between the 12th and 18th weeks in hair growth and bald areas parameters, the appearance of new hairs between the 18th and 24th weeks, the hair loss between the 12th and 18th, 12th and 24th, and 18th and 24th weeks and patient satisfaction parameter between the 12th and 18th weeks.

**Table 6:** Comparison of patients’ self-assessment within each group over 24 weeks of treatment

Cetirizine + minoxidil group *N* = 27							
Patients self-assessment parameter	Baseline	6 weeks	12 weeks	18 weeks	24 weeks	*X*^2^-value	*P*-value* (< 0.05)
	Mean rank	Mean rank	Mean rank	Mean rank	Mean rank		
Hair growth	1.30	2.43	3.22	3.74	4.31	81.662	< 0.0005*
New hairs	1.07	2.43	3.26	3.87	4.37	90.590	< 0.0005*
Bald areas	4.91	3.52	2.81	2.33	1.43	88.167	< 0.0005*
Hair loss	4.98	3.30	2.54	2.17	2.02	90.100	< 0.0005*
Satisfaction	1.09	2.70	3.39	3.59	4.22	86.456	< 0.0005*

Placebo + minoxidil group *N* = 30							
Hair growth	1.23	2.62	3.25	3.80	4.10	89.021	< 0.0005*
New hairs	1.08	2.47	3.37	3.97	4.12	98.200	< 0.0005*
Bald areas	4.97	3.48	2.70	2.03	1.82	98.507	< 0.0005*
Hair loss	5.00	3.12	2.68	2.20	2.00	96.121	< 0.0005*
Satisfaction	1.08	2.95	3.40	3.68	3.88	88.835	< 0.0005*

^*^Friedman test (*P* < 0.05, exact significance, 2-tailed)

In group 2, all parameters also significantly improved over time except for the following: there was a non-significant difference in patients’ responses to the hair growth between the 6th and 12th weeks, and 18th and 24th weeks, the new hairs appearance between the 12th and 18th weeks and between the 18th and 24th weeks, the bald areas between the 18th and 24th weeks, the hair loss between the 6th and 12th, 12th and 18th, and 18th and 24th weeks and patient satisfaction parameter between the 6th and 12th, 6th and 18th, 12th and 18th, 12th and 24th and 18th and 24th weeks.

### Global photographic assessment

The blinded outcome assessor (mean ranks: group 1 = 30.05 and *N* = 28, group 2 = 28.98 and *N* = 30) and the blinded dermatologists panel (mean ranks: group 1 = 29.68 and *N* = 28, group 2 = 29.33 and *N* = 30) responses to the degree of patients improvement from baseline were compared between the 2 groups at 12 weeks using Mann–Whitney *U* test, exact significance and there was no statistically significant difference (*P* = 0.772 and 0.941, respectively). At 24 weeks, the improvement from baseline according to the outcome assessor (mean ranks: group 1 = 29.40 and *N* = 26, group 2 = 25.73 and *N* = 28) and the blinded dermatologists panel (mean ranks: group 1 = 27.90 and *N* = 26, group 2 = 27.13 and *N* = 28) was compared between groups and there was no statistically significant difference (*P* = 0.330 and 0.859, respectively). However, according to the mean ranks, group 1 was given slightly higher scores. The blinded outcome assessor and the blinded dermatologists panel responses differed significantly over time within each group (*P* < 0.0005 and = 0.004, respectively, Wilcoxon test, exact significance) (*N* group 1 = 26, *N* group 2 = 27).

The response rates to treatment based on the panel of dermatologists were measured where slight increase in hair growth = good response, moderate increase = very good and great increase = excellent. Group 1 showed excellent response in 16 patients, very good in 7, good in 2 and no change in one patient, while in group 2, 17 patients showed excellent response, 6 were very good, 4 were good and one patient showed no change. Fisher’s exact showed statistically insignificant difference between groups.

### Treatment safety

The reported side effects were itching, dry hair, initial hair loss and dandruff which are known side effects of minoxidil and the patients did not complain of any side effects after cetirizine application. There was no significant difference between groups in the number of patients with side effects (*P* > 0.05).

## Discussion

Minoxidil is the only FDA-approved treatment for FAGA; it needs lifelong application and has side effects [10–12]. PGs have a role in hair growth where PGE and PGF increase hair growth, while PGD2 inhibits it and increases sebaceous hyperplasia and is elevated in bald scalp [3, 4]. Cetirizine increases PGE, inhibits PGD2 and reduces inflammatory cell infiltrate which makes it a potential safe treatment for AGA [13–15]. To the best of our knowledge, this is the first study on cetirizine as an add-on in FAGA.

In this study, after 24 weeks of treatment, there was a significant increase in the terminal and vellus hair density in both the frontal and vertex areas within both treatment groups. Since the increase in hair density was significant in both groups, it will be hard to know if cetirizine had a role in it. However, a short report by Rossi et al. in patients with congenital hypotrichosis caused by ectodermal dysplasia [24] and a case report of a 70-year-old woman with chemotherapy-induced alopecia [25] showed positive effect of topical cetirizine on hair density.

The hair shaft thickness significantly increased between baseline and the 12th week in group 1 only in the vertex area only. This may be because in FAGA usually the vertex area is the most affected while the frontal hair line is kept intact [26], so the patients may have used more puffs on the vertex area. Although the increase in hair density was high compared to the increase in thickness, other studies showed similar results with modest increase in thickness after using minoxidil 5% twice daily [16, 27, 28].

The average number of hairs per follicular unit increased significantly between baseline and the 12th week in both areas in group 1. It also increased insignificantly in group 2.

A preliminary study by Rossi et al. [17] on 85 males and females, but the number of females was unclear and no subgroup analysis by gender was performed, compared the effect of cetirizine versus placebo after 6 months. The study showed a significant increase in terminal hair density and hair shaft diameter in the cetirizine group. These results are in line with this study results, where there was a significant increase in terminal hair density within both groups and a significant increase in the hair shaft thickness in the vertex within the cetirizine group. However, Rossi et al. [17] reported a decrease in vellus hair density, while in this study, the vellus hair density significantly increased in both areas in both groups. This was partially explained by the idea that minoxidil at first promotes vellus hair production, and then, these vellus hairs are turned into terminal hairs by continuing treatment. Therefore, the increase in total hair density depends mainly on the growth of vellus hairs rather than terminal hairs [16].

Another study by Mostafa et al. [16] was conducted on 40 males, to compare cetirizine versus minoxidil after 16 weeks of treatment. That study showed slight non-significant increase in terminal hair density and a significant increase in vellus hair density within and between groups, but the improvement was higher in the minoxidil group. There was no significant change in hair diameter in any group.

In this study, regarding the patients’ self-assessment, by the 24th week, group 1 gave significantly higher scores to the clinical improvement in all parameters except for the hair loss parameter that was insignificantly different between groups at all the time points because minoxidil decreases hair fall. The parameter of the appearance of new hairs was significantly better in group 1 at the 12th and 18th weeks, so, cetirizine may help the growth of new baby hairs from patients’ perspective. Another study conducted on 60 males showed better patient satisfaction with cetirizine compared to placebo [15] while Mostafa et al. [16] showed better patient satisfaction with minoxidil compared to cetirizine. Both studies asked the patients one question to assess their overall improvement using a 6-point [15] and a 5-point scale [16].

The outcome assessor and the panel of dermatologists gave group1 better scores, but it was insignificantly different from group 2. This was comparable to other studies where physicians observed higher increase in hair density in the cetirizine group compared to minoxidil using a 5-point scale [16] and higher increase in hair density in the cetirizine group compared to placebo using a 4-point scale [15].

Regarding safety, transient side effects of minoxidil were reported. None of the patients reported a specific side effect after cetirizine application. The percent of patients who reported side effects was insignificantly different between groups. This aligns with the safety profile of cetirizine and with previous studies [15–17]. Cetirizine has anti-inflammatory and anti-allergic properties so it has lower side effects than minoxidil.

Limitations of this study included the absence of a cetirizine arm because by the time the study started there was only one paper about the efficacy of topical cetirizine in AGA. Therefore, we did not want to cause harm by depriving the patients from the standard of care without having enough evidence which may have diminished the effect size of cetirizine [29]. Although trichoscopic parameters were measured using phototrichoscopy, patients’ hair was undyed and unclipped. However, smart count mode was used and allowed to measure white hair count and diameter. Clipping hair is not usually done at the clinic due to patients’ resistance based on cultural background and psychological impact of their appearances.

In conclusion, cetirizine may clinically improve FAGA and increase shaft thickness with no side effects which makes it a potential therapy for FAGA. Pharmacokinetic studies on its bioavailability in follicular hair sheath and dose-dependent hair growth are recommended along with studies to assess the exact mechanism of action of the drug, its effectiveness in other alopecia types, the efficacy of its oral dosage form and other H1-blockers.

## Data Availability

Data are available on request from the authors.
